# Hybrid histidine kinases shape the architecture of the high osmolarity glycerol (HOG) pathway: from *Saccharomyces cerevisiae* to the priority pathogen *Aspergillus fumigatus* and other filamentous fungi

**DOI:** 10.1093/femsre/fuag037

**Published:** 2026-08-03

**Authors:** Sebastian Schruefer, Yanning Hu, Frank Ebel

**Affiliations:** Chair of Bacteriology and Mycology, Institute for Infectious Diseases and Zoonoses, Ludwig-Maximilians-University Munich, D-85764 Oberschleissheim, Munich, Germany; Chair of Bacteriology and Mycology, Institute for Infectious Diseases and Zoonoses, Ludwig-Maximilians-University Munich, D-85764 Oberschleissheim, Munich, Germany; Chair of Bacteriology and Mycology, Institute for Infectious Diseases and Zoonoses, Ludwig-Maximilians-University Munich, D-85764 Oberschleissheim, Munich, Germany

**Keywords:** HOG pathway, hybrid histidine kinase, multistep phosphorelay, fungal stress response, Ypd1

## Abstract

The high osmolarity glycerol (HOG) pathway enables adaptation to hyperosmotic and other environmental stresses. This review highlights key differences between the pathway in *Saccharomyces cerevisiae* and in filamentous fungi, focusing on the major pathogen *Aspergillus fumigatus*. In the multistep phosphorelay of the HOG pathway, stress signals are detected by hybrid histidine kinases (HHKs). According to the current model, HHKs function as kinases under nonstress conditions to maintain phosphorylation of downstream relay components. Upon stress exposure, their activity decreases or may switch to phosphatase activity, resulting in dephosphorylation of the relay. This change ultimately activates the downstream MAP kinase cascade, creating an inverse relationship between phosphorylation and signaling output in the two sections of the pathway. Fungi differ markedly in their HHK repertoire, ranging from a single HHK in *S. cerevisiae* to multiple functionally distinct HHKs in filamentous fungi. This review examines how this diversity has shaped the organization of the multistep phosphorelay and its integration into the HOG pathway. We summarize current knowledge of HHK function across fungal species, discuss why the multistep phosphorelay represents an Achilles’ heel for fungi, and consider how the spatial organization of HOG pathway components influences signaling and how HHK repertoires may have diversified across the fungal kingdom.

## Teaser

Impact of hybrid histidine kinases on the organization of the high osmolariry glycerol pathway in filamentous fungi.

Histidine kinases are versatile signaling molecules distributed across diverse domains of life. Although rare in mammals, they are widespread in bacteria, archaea, slime molds, plants, and fungi (Besant and Attwood 2005, Hoang et al. 2021). As core components of two-component systems, histidine kinases play a central role in sensing and transducing environmental cues. In fungi, canonical two-component signaling has evolved into hybrid histidine kinases (HHKs), which are unique to this kingdom and constitute a key element of the fungal signaling machinery (Bahn 2008). HHKs are multidomain proteins that enable fungi to perceive and integrate a broad range of external and intracellular signals, thereby coordinating stress adaptation, growth, and development (Defosse et al. 2015).

The high osmolarity glycerol (HOG) pathway is one of the major fungal stress response pathways (Bahn et al. 2007) and involves HHKs that function as sensor proteins. Whereas *Saccharomyces cerevisiae* contains only a single HHK, filamentous fungi possess multiple HHKs that form a regulatory network controlling HOG activity through Ypd1 phosphorylation. In contrast to the less complex signaling system of *S. cerevisae*, this arrangement creates a considerably more complex signaling architecture, capable of integrating diverse environmental cues into appropriate adaptive responses.

The name-defining function of the HOG pathway is to enable adaptation to a hyperosmotic environment. An increased osmotic pressure in the external milieu forces microorganisms to adjust their internal osmotic pressure to maintain water balance and cellular volume. The HOG pathway initiates and controls this adaptive process. However, the relevance of this pathway extends far beyond osmoadaptation. As a primary stress response system, the HOG pathway integrates a wide range of signals, including oxidative, thermal, cell wall stress, and light sensing, thereby functioning as a central hub for environmental adaptation in fungi (Bahn et al. 2007, Yaakoub et al. 2022, Leister et al. 2025).

The basic architecture of the HOG pathway was originally defined in the model organism *S. cerevisiae* (Hohmann 2009) and was long assumed to be broadly conserved across the fungal kingdom. However, a fundamental organizational difference distinguishes *S. cerevisiae* from most other fungi and has important implications for the organization of the pathway. Whereas *S. cerevisiae* encodes only a single HHK, filamentous fungi harbor multiple HHKs, of which an as-yet undefined subset feeds into the multistep phosphorelay. This relay, comprising at least four proteins, represents a key element of the HOG pathway and links HHKs to downstream response regulators via the histidine phosphotransfer (Hpt) protein Ypd1. Although composed of only few components, the multistep phosphorelay exhibits a considerable high level of functional complexity. The individual contributions of different HHKs to this signaling hub remain a crucial but incompletely understood aspect of pathway regulation.

Before addressing these aspects in more detail, we will summarize the studies that established our current understanding of the HOG pathway in *S. cerevisiae*.

## The HOG pathway in *S. cerevisiae*: paradigm and exception

The foundational work was done by Brewster et al. (1993), who identified the mitogen-activated protein kinase (MAPK) Hog1p and its upstream MAP kinase kinase (MAPKK) Pbs2p as central components of osmoadaptation. Subsequent studies revealed that the HOG pathway exhibits a branched architecture. Two distinct membrane-associated sensors, Sln1p and Sho1p, initiate separate upstream signaling pathways that converge at the level of Pbs2p (Brewster and Gustin 2014).

The Sho1p branch functions through a structurally complex system. Under high osmolarity, conformational changes promote binding of Sho1p to the adaptor protein Ste50p, which in turn activates further components of the HOG pathway (Tatebayashi et al. 2015). Sho1p also interacts with the mucin-like transmembrane proteins Msb2p and Hkr1p, which sense environmental signals, recruit the MAPKKK Ste11p and the MAPKK Pbs2p and finally activate the MAPK Hog1p (Tatebayashi et al. 2007, Saito and Posas 2012).

In the Sln1p branch, the membrane-bound HHK Sln1p transfers phosphoryl groups to the soluble Hpt protein Ypd1p to keep the pathway in an inactive state. This constitutive kinase activity in the absence of stress is a hallmark of the Sln1p branch. An increase of the osmotic pressure in the environment reduces the relative intracellular turgor pressure. As a consequence, the cellular body shrinks and the plasma membrane retracts from the cell wall. The kinase activity of Sln1p depends on the contact between its extracellular domain and the cell wall; a loss of this contact abrogates Sln1p’s kinase activity and leads to the activation of the HOG pathway (Reiser et al. 2003).

In the absence of stress, phosphorylated Ypd1p relays phosphoryl groups to the response regulators Ssk1p and Skn7p. Although Skn7p is not a component of the canonical HOG pathway, it constitutes an integral branch of the multistep phosphorelay upstream of the HOG MAP kinase cascade and is therefore functionally and mechanistically closely linked to HOG signaling. Moreover, in several filamentous fungi, Skn7 has been shown to contribute to adaptation to hyperosmotic stress (Schruefer et al. 2021a, Motoyama et al. 2008, Oide et al. 2010, Chen et al. 2012). For these reasons, we consider Skn7 to be part of the HOG signaling network in a broader sense.

The MAP kinase module downstream of Ssk1p includes the MAPKKKs Ssk2p (Posas and Saito 1998, Li et al. 1998) and Ssk22, which are redundant in terms of function (Maeda et al. 1995). The activity of both proteins is tightly regulated by Ssk1p; in its phosphorylated stage, Ssk1p is inactive and unable to bind to either Ssk2p or Ssk22. The continuous flow of phosphoryl-groups through the Sln1p–Ypd1p–Ssk1p axis therefore maintains the pathway in an inactive state.

If hyperosmotic stress is encountered, the flow of phosphoryl groups is stalled. As a consequence, Ssk1p becomes dephosphorylated and able to bind to Ssk2p and Ssk22p. Within the complex, the MAPKKKs are autophosphorylated, which initiates the MAP kinase cascade (Posas and Saito 1998). Like many MAPKKKs, Ssk2/Ssk22 possess a catalytic domain in their C-terminal and an auto-inhibitory domain in their N-terminal parts. By binding, Ssk1 induces a conformational change that relieves the catalytic domain of Ssk2p from inhibition by the autoinhibitory domain, which allows autophosphorylation of Ssk2p (Posas and Saito 1998).

**Figure 1:**
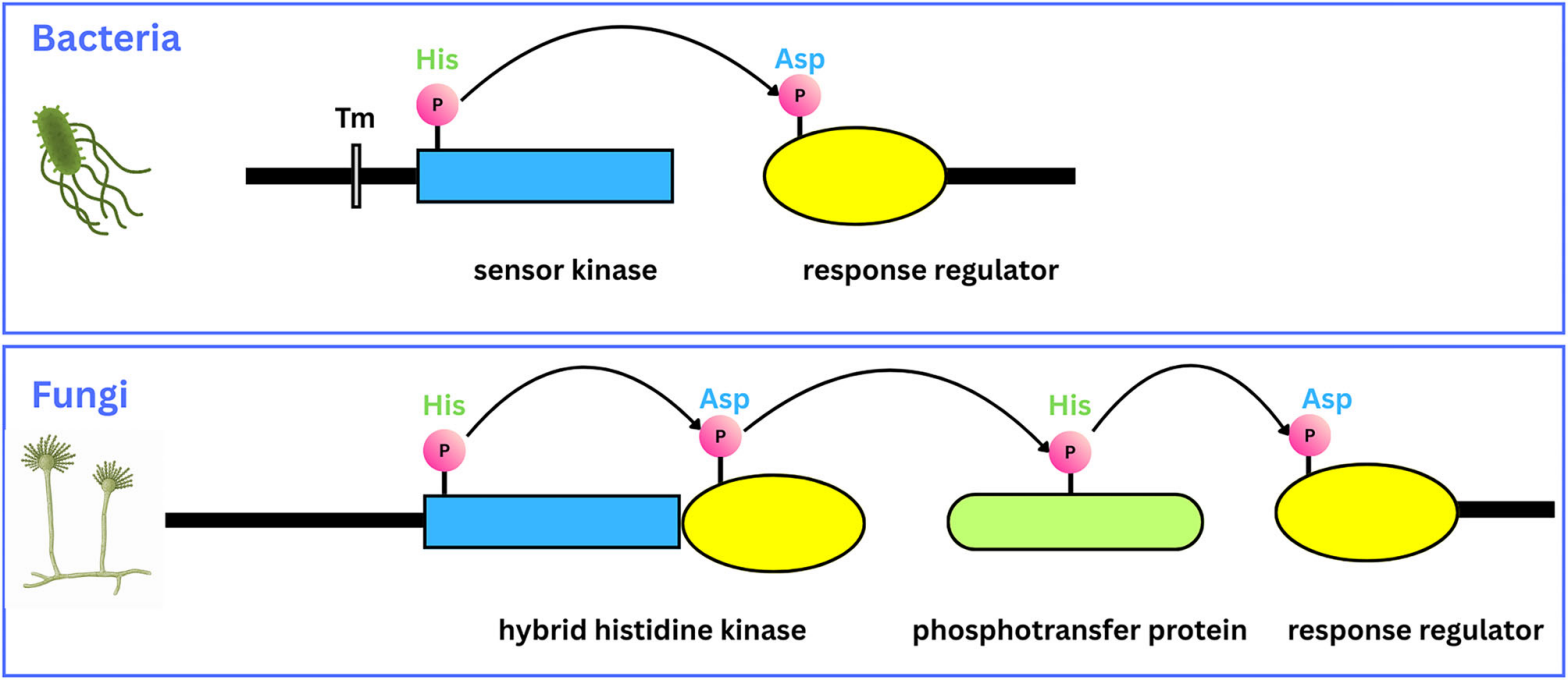
Two-component signaling in bacteria and fungi. Phosphotransfer reactions are indicated by arrows. His = histidine residue. Asp = aspartate residue. Tm = transmembrane region

The multistep phosphorelay evolved from bacterial two-component systems (Posas et al. 1996) that typically consist of a membrane sensor histidine kinase and a response regulator protein. In response to an external signal, the kinase autophosphorylates a conserved histidine (His) residue in its DHp (dimerization and histidine phosphotransfer) domain and this phosphoryl group is then transferred to a conserved aspartate (Asp) residue in the receiver (REC) domain of the response regulator (Fig. 1).

In fungi, both elements of the two-component system have been integrated into a single hybrid molecule. A typical example for this architecture is Sln1p; it becomes autophosphorylated at a conserved His residue of its histidine kinase A domain (HisKA) domain. The phosphoryl group is subsequently transferred to an Asp residue in the REC domain of Sln1p and then to a His residue of the Hpt protein Ypd1 (Fig. 1). Ypd1p is small (<20 kDa) and can freely travel through the nuclear pore. While shuttling between the cytoplasm and the nucleus, it can transfer phosphoryl groups to Asp residues in the two response regulators of *S. cerevisiae*: the cytoplasmic Ssk1p and the nuclear Skn7p (Lu et al. 2003). Overall, the transfer sequence follows the pattern His → Asp → His → Asp, which defines the characteristic sequence of a fungal multistep phosphorelay (Fig. 1).

**Figure 2:**
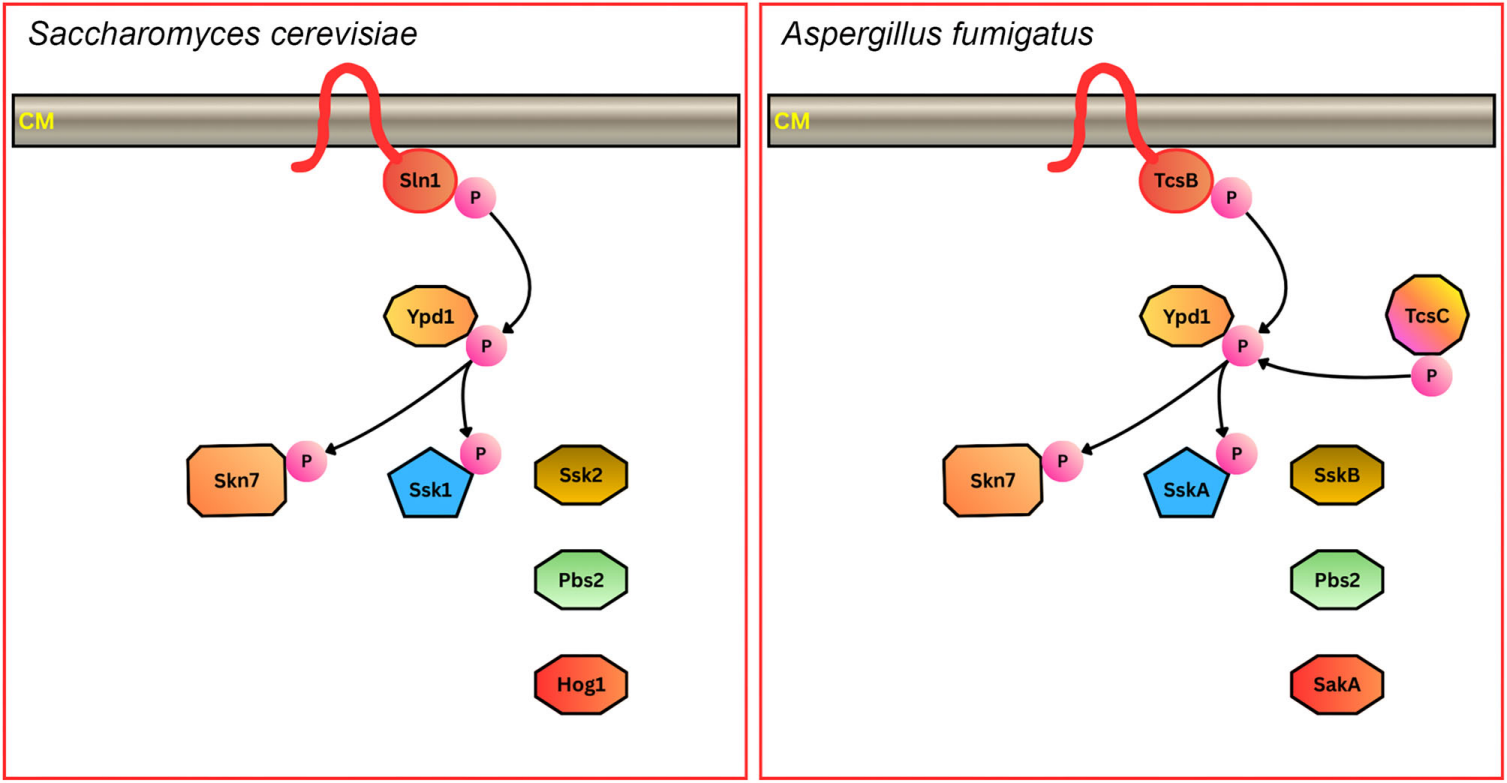
The HOG pathway in *S. cerevisiae* and *A. fumigatus* in the absence of stress. The phosphorylated proteins and the phosphotransfer reactions are indicated. The scheme summarizes similarities and differences in nomenclature of the HOG proteins in these two fungi. CM = cytoplasmic membrane.

In the absence of stress, Sln1p continuously phosphorylates Ypd1p and, in turn, Ssk1p, to keep the MAP kinase module in an inactive state. This situation is depicted in Fig. 2 for *S. cerevisae* and *Aspergillus fumigatus*. The model also highlights similarities and difference in the nomenclature used in both fungi to designate the individual proteins. Upon hyperosmotic stress, the kinase activity of Sln1p is inhibited and phosphotransfer is stalled. As a result, Ssk1p becomes dephosphorylated and activates Ssk2p and the MAP kinase cascade, which in turn leads to a differential expression of genes involved in osmo-adaptation and to a Hog1p-driven activation of glycerol production to restore the osmotic balance (Posas and Saito 1998).

In the cytoplasm, Ssk1p exists predominantly as a dimer, and genetic analyses have shown that efficient activation of the MAPKKK Ssk2p requires interaction with a dimer composed of two unphosphorylated Ssk1p molecules (Horie et al. 2008). This requirement reflects a stringent regulatory mechanism: only when the majority of Ssk1p is dephosphorylated can Ssk2p be efficiently activated. To allow a rapid response to osmotic stress, inhibition of Ypd1p-mediated phosphotransfer must therefore be accompanied by fast dephosphorylation of Ssk1p. Consequently, Ssk1p phosphorylation is expected to be highly transient to permit a rapid response to hyperosmotic stress.

Biochemical analyses demonstrated that phosphorylated Ssk1p is indeed intrinsically unstable, with a half-life of ~13 min. In intact cells, however, Ssk1p phosphorylation is markedly prolonged, exhibiting a half-life of roughly 42 h. This stabilization depends on the binding of Ypd1p to phospho-Ssk1p, which shields the aspartyl phosphate from hydrolytic decay (Janiak-Spens et al. 1999, 2000). These observations imply that rapid signaling responses must involve mechanisms that actively destabilize phosphorylated Ssk1p. One possibility is the existence of a dedicated phosphatase; however, no such enzyme has been identified to date. Alternatively, dephosphorylation of Ypd1p by an upstream component of the HOG pathway could reverse the directionality of phosphotransfer, promoting back-transfer of the phosphoryl group and thereby accelerating Ssk1p dephosphorylation. Such a mechanism would provide a dynamic means to overcome Ypd1-mediated stabilization and enable rapid pathway activation. This topic is discussed in greater detail in a later section of this review.

MAP kinase cascades are evolutionarily conserved signaling units found in diverse eukaryotic organisms, including fungi and yeast (Chen et al. 2001), but their upstream elements are more variable, particularly at the level of proteins involved in signal perception.

Adaptive responses are typically transient. Once homeostasis is restored, activated signaling components return to their basal activity state. Phosphatases play an important role in the regulation of the MAP kinase cascade. For Pbs2p this is mediated by Ptc1–Ptc4 (Tatebayashi and Saito 2023), whereas Hog1p is the target of the protein tyrosine phosphatases Ptp2p and Ptp3 (Wurgler-Murphy et al. 1997).

## The multistep phosphorelay—a unique signaling hub

An important conceptual distinction exists between the multistep phosphorelay and the downstream MAP kinase cascade, both of which have been extensively studied in *S. cerevisiae*. In the multistep phosphorelay, phosphorylation serves to suppress the stress response by maintaining Ssk2p in an inactive state, whereas in the MAP kinase cascade, phosphorylation of Ssk2p induces signaling and transcriptional activation. The interaction between Ssk1p and Ssk2p/Ssk22p therefore functions as a molecular switch that inverts the relationship between phosphorylation and cellular outcome. The distinction between the two parts of the pathway is also reflected at the level of the phosphorylated residues. In the multistep phosphorelay, phosphoryl groups travel from His to Asp, while components of the MAP kinase cascade are phosphorylated at threonine and serine residues and in the case of the MAPK also at tyrosine residues (Maeda et al. 1995, Posas and Saito 1998, Saito and Posas 2012, Tatebayashi et al. 2020).

Two aspects of the multistep phosphorelay are particular remarkable:

First, its strict requirement for negative regulation, since any disruption in the control mechanism can lead to activation of the MAP kinase module, causing excessive water influx and osmotic imbalance, often with fatal consequences. Accordingly, SLN1 or YPD1 deletions are lethal in *S. cerevisiae* (Maeda et al. 1994, Posas et al. 1996), whereas SSK1 is dispensable, reflecting the distinct position of Ssk1p within the regulatory framework.

Second, the architecture of the multistep phosphorelay appears counterintuitive from an energy-efficiency perspective, as it requires energy in the absence of stress. The HOG pathway can be compared to “a car that consumes fuel while parked but not while driving.” This design likely confers an evolutionary advantage, although the nature of this benefit is unknown. Interestingly, bacterial two-component systems, the evolutionary ancestors of the fungal multistep phosphorelay systems, differ in that they remain inactive until stress is encountered and the same applies to the Sho1p branch of the HOG pathway (Stock et al. 2000, Tatebayashi et al. 2015).

## The HOG pathway in *A. fumigatus*

In *A. fumigatus*, the HOG pathway is a major regulator of stress adaptation and a target of antifungal agents such as fludioxonil. Compared with *S. cerevisiae*, its overall organization is well conserved (Figs 2 and 4). Azuma et al. (2007) demonstrated that *Aspergillus* species employ the same multistep phosphorelay mechanism as *S. cerevisiae*. Subsequently, Furukawa et al. (2005) identified and functionally characterized the core components of the *A. nidulans* HOG MAP kinase cascade, namely SskA, SskB, PbsB, and SakA (Fig. 2).

**Figure 3:**
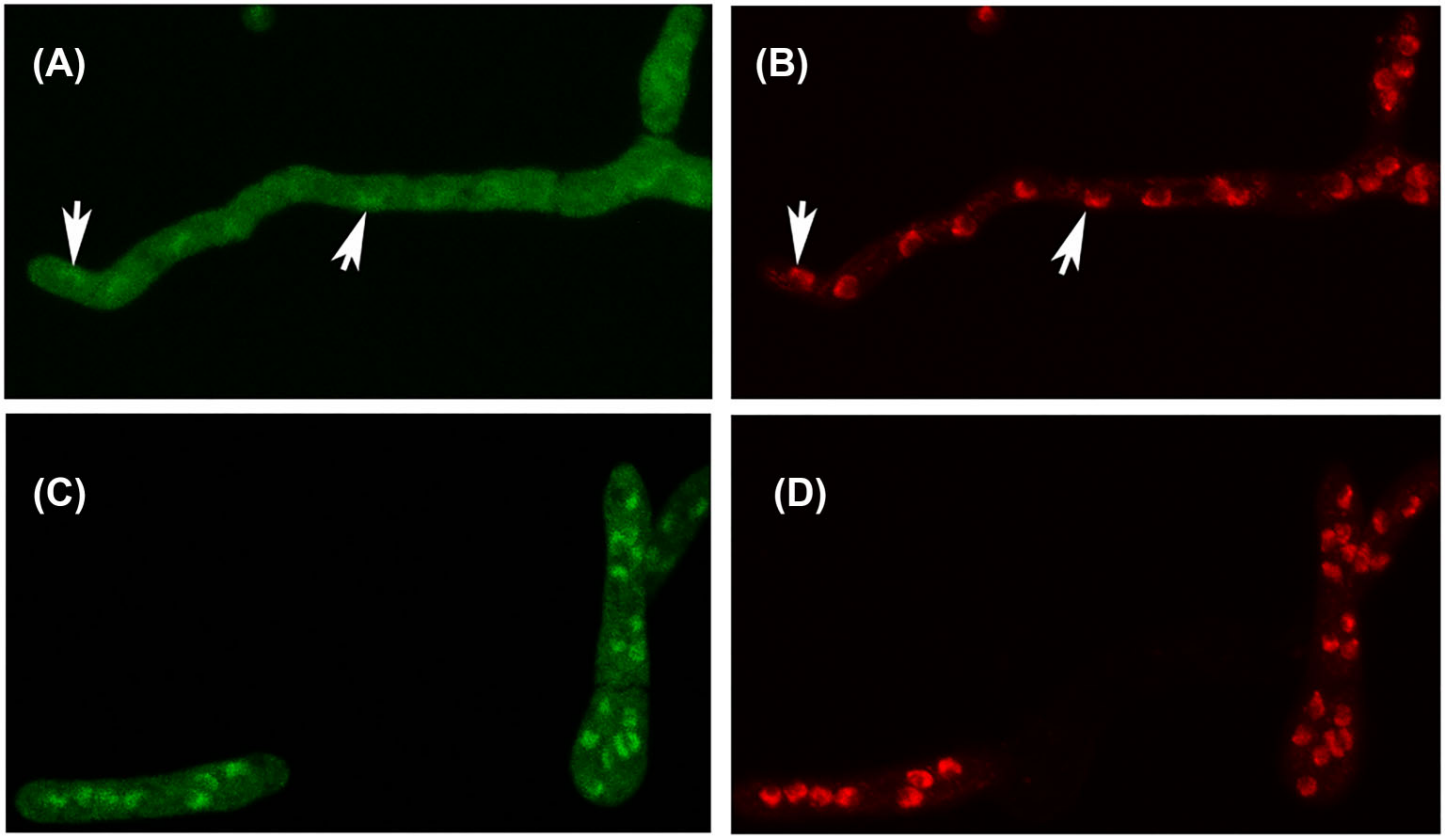
Basal activation of SakA in *A. fumigatus* hyphae in the absence of stress. Localization of SakA-GFP before and 3 h after treatment with fludioxonil (1 µg/ml) is shown in A and C, respectively. DAPI (shown in false colour) was used to detect nuclei (B and D). Note that in the absence of stress, small amounts of SakA-GFP are already recruited to the nuclei (A and B, arrows).

SakA is the *Aspergillus* ortholog of the *S. cerevisiae* MAP kinase Hog1p. Macia et al. (2009) demonstrated that Hog1p displays substantial basal activity and proposed that this constitutive activity enables a more dynamic and responsive regulation of the HOG pathway. Phosphorylation of both Hog1p and its *Aspergillus* ortholog SakA triggers their translocation to the nucleus (Ferrigno et al. 1998, Lara-Rojas et al. 2011). In *A. fumigatus*, a small proportion of SakA is detectable in the nucleus even in the absence of stress, whereas hyperosmotic stress or exposure to the antifungal fludioxonil markedly increases its nuclear accumulation (Fig. 3). The presence of nuclear SakA under nonstress conditions is consistent with the basal activity reported for Hog1p and suggests that constitutive, low-level activation of the HOG pathway may be a conserved feature of fungi. One possible explanation for this basal activity is inadvertent exposure to light during cultivation or microscopy, as light has been shown to activate SakA (Yu et al. 2019).

**Figure 4:**
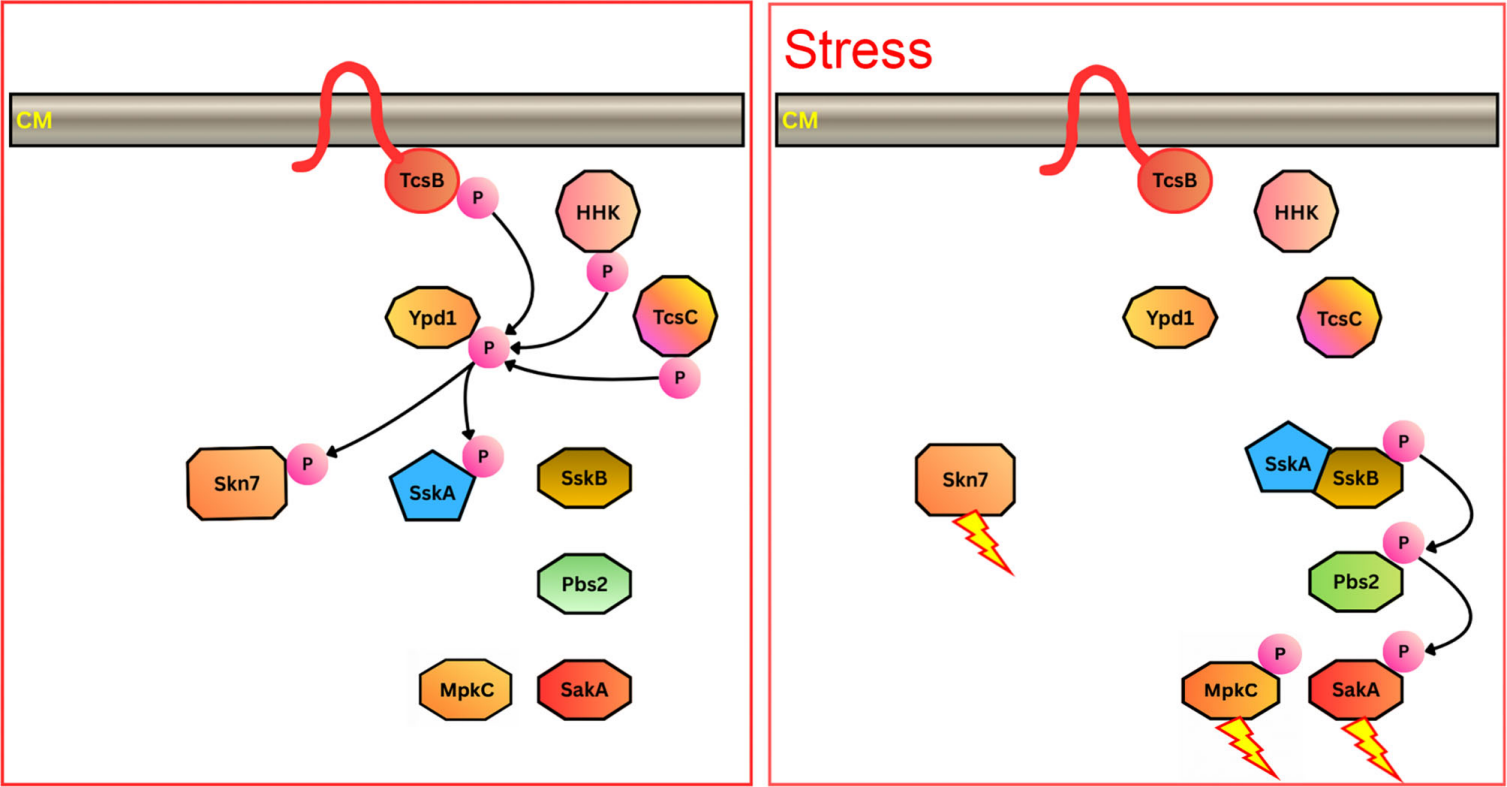
Model of the HOG pathway of *A. fumigatus* in the presence and absence of stress. Activation of SakA and Skn7 is indicated by a flash-like symbol. Note that the role of HHKs, including TcsB, in the phosphorylation of Ypd1 is still not proven experimentally. CM = cytoplasmic membrane.

Compared with *S. cerevisiae*, the *Aspergillus* HOG pathway differs in two important aspects. First, whereas baker’s yeast possesses two MAPKKKs (Ssk2p and Ssk22p), *Aspergillus* species harbor a single HOG pathway MAPKKK, SskB (Furukawa et al. 2005, Hagiwara et al. 2014). Second, *Aspergillus* genomes encode four MAPKs, two of which, SakA and MpkC, are Hog1 g homologs and operate downstream of the MAPKK PbsB (Furukawa et al. 2005, Day and Quinn 2019).

The initial characterization of a Δ*mpk*C mutant revealed only a defect in polyalcohol utilization and little contribution to osmotic stress resistance (Reyes et al. 2006). Hagiwara et al. (2014) later confirmed that MpkC plays only a minor role in the response to hyperosmotic stress. However, they also showed that MpkC assumes a more prominent role in other stress responses, acting redundantly with SakA to confer resistance of conidia to thermal and oxidative stress.

Bruder Nascimento et al. (2016) showed that SakA and MpkC accumulate in the nucleus of *A. fumigatus* in response to hyperosmotic stress and the antifungal drug caspofungin. The two MAPKs furthermore interact physically under hyperosmotic and cell wall stress (Manfiolli et al. 2019), suggesting that they cooperate in coordinating the stress response. Taken together, these studies indicate that MpkC is activated in response to hyperosmotic stress and physically interacts with SakA. Although SakA is the predominant regulator of osmoadaptation, MpkC may contribute to this stress response through partially overlapping functions.

In *A. fumigatus*, loss of either SakA or MpkC had no significant impact in mouse survival experiments, whereas the Δ*mpk*CΔ*sak*A double mutant was severely attenuated in virulence (Bruder Nascimento et al. 2016). This implies that both MAP kinases play redundant roles during infection. The strongly reduced stress resistance of Δ*mpk*CΔ*sak*A conidia (Hagiwara et al. 2014) is likely to contribute to the attenuated virulence of the double mutant.

In the MAP kinase cascade of the HOG pathway, phosphoryl groups are transferred from SskB via PbsB to either SakA or MpkC (Fig. 4). Notably, conidia of a Δ*ssk*A mutant were more sensitive to stress than those of the Δ*sak*A and Δ*mpk*C single mutants (Hagiwara et al. 2014) and SskA, that functions upstream of both SakA and MpkC, has been shown to play an important role during germination and disease progression in pulmonary infections (Kirkland et al. 2021). Additional evidence for the importance of the HOG pathway during infection comes from the analysis of clinical isolates. A strain of *A. fumigatus* recovered from the lung of a patient with cystic fibrosis (CF) carried a mutation in *pbsB* (*pbs2*) that conferred enhanced adaptation to the hyperosmotic and hypoxic conditions characteristic of the CF lung. However, this adaptive advantage was accompanied by reduced conidial stress resistance under normoxic conditions, indicating a trade-off between host-specific adaptation and environmental stress tolerance (Ross et al. 2021).

Collectively, these findings establish the HOG pathway as a major determinant of *A. fumigatus* pathogenicity, most likely through its central role in coordinating adaptation to the diverse stresses encountered during infection.

## The repertoire of HHKs in *Aspergillus* and other filamentous fungi

Sln1p is the sole sensor of the HOG pathway in *S. cerevisiae*. In contrast, most other fungi, in particular filamentous species, harbor multiple HHKs. Despite their diversity, all HHKs contain a conserved C-terminal signaling module consisting of three domains that cooperate: a histidine kinase-like ATPase domain (HATPase-c) autophosphorylates a conserved His residue in the HisKA domain, followed by transfer of the phosphoryl group to a conserved Asp residue in the most C-terminal REC domain (Fig. 1).

This conserved C-terminal architecture contrasts with a highly variable N-terminal part and these differences have been used to classify HHKs in different groups. Catlett et al. (2003) defined 11 groups based on their N-terminal domain architecture and this number was later on expanded to 19 (Defosse et al. 2015, Kabbara et al. 2019). Sln1p is the prototypical member of group VI, whereas other *Saccharomycotina* possess HHKs belonging to groups III, V, VI, X, and XI (Hérivaux et al. 2018). Even larger numbers of HHKs are found in many filamentous Ascomycota, whereas a smaller variability is characteristic for filamentous fungal pathogens belonging to the Basidiomycota or Mucorales (Defosse et al. 2015).

Although HHKs are generally regarded as sensor proteins, all of them, except for group VI, are localized in the interior of the cell, whereas their bacterial counterparts are typically embedded in the cytoplasmic membrane and thereby able to directly interact with the external environment (Stock et al. 2000). Group VI HHKs possess two transmembrane regions that divide the protein into extracellular and intracellular parts. Apart from the two membrane-spanning regions, group VI HHKs have no detectable domains in their N-terminal sensing modules, which is another feature that distinguishes them from all other HHK groups.

Members of several HHK groups harbor PAS or GAF domains (Table 1), but to date little is known on the function of these proteins (Defosse et al. 2015). The name PAS (Per–Arnt–Sim) derives from the three proteins in which this domain was first identified. PAS domains are located in the N-terminal regions of a wide range of signaling proteins and share a conserved three-dimensional fold that enables binding of specific ligands (Henry and Crosson 2011). GAF domains adopt a three-dimensional fold similar to that of PAS domains and commonly bind cyclic guanosine monophosphate (Ho et al. 2000).

**Table 1:** The HHKs of *A. fumigatus*.

Group	Systematic name	Designation	Domains and features*	Dimer interface**
I	Afu4g01020	Fhk1	GAF domain	Yes
I	Afu3g07130	Fhk2	GAF domain	Yes
I	Afu8g06140	Fhk3	GAF domain	Yes
I	Afu4g00660	Fhk5	GAF domain	Yes
I	Afu4g00320	Fhk6	GAF domain	Yes
III	Afu2g03560	TcsC	Six HAMP domains	Yes
IV	Afu6g10240	TcsA/Fos-1	PAS domain	Yes
V	Afu3g12530	PhkB	Two PAS and two PAC domains	Yes
VI	Afu2g00660	TcsB	Two transmembrane regions	No
VII	Afu4g07400	Fhk4	GAF domain	No
VIII	Afu4g02900	FphA	PAS_2, GAF, and SCOP 8025074 domains	Yes
VIII	Afu6g09260	FphB	GAF and PHY domains	Yes
X	Afu3g12550	PhkA	GAF, SCOP 8062796, and PfamAAA_16 domains	Yes

Protein designations were obtained from FungiDB and Chapeland-Leclerc et al. (2015).

* Predicted by SMART;

** predicted by InterProScan.

Phytochromes in plants, bacteria, and fungi typically contain light-sensing modules composed of phytochrome, PAS and GAF domains. In *Aspergillus*, the group VIII HHKs FphA and FphB have been implicated in the sensing of light and temperature, which also involves the HOG pathway and the group VI HHK TcsB (Blumenstein et al. 2005, Froehlich et al. 2005, Yu et al. 2019). Moreover, FphA physically interacts with the global regulator VeA to repress sexual development in the presence of red light (Blumenstein et al. 2005, Purschwitz et al. 2009). This interaction highlights a broader regulatory role for FphA, functionally connecting light perception with the control of fungal morphogenesis.

## Functional roles of HHKs: limited but emerging evidence

In two filamentous fungi, the complete sets of HHK genes have been systematically analyzed using single deletion mutants. Chapeland-Leclerc et al. (2015) generated single-gene deletion mutants for all 13 HHK-encoding genes in *A. fumigatus*, a mold classified by the World Health Organization as belonging to the Critical Priority Group (WHO 2022). These HHKs belong to eight different groups, namely group I (Fhk1, Fhk2, Fhk3, Fhk5, and Fhk6), III (TcsC), IV (TcsA), V (PhkB), VI (TcsB), VII (Fhk4), VIII (FphA and FphB), and X (PhkA) (Table 1). Functional analyses implicate Fhk6 and PhkA in the regulation of the “fluffy” developmental program; PhkA is involved in the oxidative stress response and PhkB plays a role in conidiation. The most striking phenotype was observed for the mutant lacking the group III HHK TcsC. This mutant was highly sensitive to hyperosmotic stress and completely resistant to dicarboximide and phenylpyrrole antifungals (Chapeland-Leclerc et al. 2015). These traits, along with an increased resistance to the cell wall stressors Congo red and Calcofluor white, were also reported in the initial characterizations of *A. fumigatus* Δ*tcs*C mutants (McCormick et al. 2012, Hagiwara et al. 2013).

In the second systematic study, Jacob et al. (2014) analysed the 10 HHKs of the plant pathogenic fungus *Magnaporthe oryzae*. Two of them, namely MoHik5p (group V) and MoHik8p (group XI), are essential for pathogenicity and loss of MoSln1p (group VI) renders the fungus more susceptible to high salt stress. Both systematic studies have in common that most mutants had a weak or no discernable phenotype, which may hint to functional redundancy.

Numerous studies have examined individual or small sets of HHK mutants in different fungi, of which only a few are highlighted here. In *A. fumigatus*, a Δ*tcs*A mutant showed enhanced resistance to dicarboximide antifungals (Pott et al. 2000) and a Δ*tcs*B mutant a pronounced sensitivity to higher temperatures (Ji et al. 2012). However, both phenotypes could not be confirmed by Chapeland-Leclerc et al. (2015). In a more recent study, loss of *tcs*B (*sln*A) rendered *A. fumigatus* more sensitive to gliotoxin, implicating TcsB in self-protection to this mycotoxin (Alves de Castro et al. 2024).

With respect to virulence, only four *A. fumigatus* HHK mutants have been investigated so far: A Δ*tcs*A (*fos*-1) mutant (group IV) was less virulent in systemically infected mice (Pott et al. 2000) and a Δ*tcs*C mutant (group III) showed wild type-like pathogenesis in cortisone-acetate treated mice (McCormick et al. 2012). In infected *Galleria mellonella* moths, virulence was increased for a Δ*fph*B and wild type-like for a Δ*fph*A mutant (Leister et al. 2025).

In *C. albicans*, it is noteworthy that double mutants lacking SLN1 (group VI) and NIK1 (Group III) could not be generated (Yamada-Okabe et al. 1999), whereas a mutant lacking Ypd1 is viable (Mavrianos et al. 2014). *Cryptococcus neoformans* contains several HHKs, but none of them belongs to group VI. Tco1 (group III) and Tco2 (a so-called dual HHK that cannot be clearly assigned to any group), interfere with the HOG pathway and play shared and distinct roles in stress responses and drug sensitivity. It is remarkable that a double mutant lacking *tco*1 and *tco*2 is viable (Bahn et al. 2006), suggesting that at least three HHKs control the activity of the HOG pathway in this pathogenic fungus. Of the 20 HHKs encoded in the genome of *Botrytis cinerea*, the group III kinase Bos1 is required for osmoregulation and contributes to virulence. It is also the target of dicarboximide and phenylpyrrole fungicides that have been used in agriculture for decades to control *B. cinerea* infections (Kilani and Fillinger 2016).

## Ypd1—the heart of the multistep phosphorelay

The high level of conservation in the signaling modules of fungal HHKs suggest that these proteins share a broadly similar mechanism and act on comparable downstream targets. Since Sln1p is known to phosphorylate Ypd1p, it is reasonable to assume that members of other HHK groups operate in a similar fashion. Genomic analyses revealed that most Ascomycetes possess only one Hpt protein commonly designated Ypd1 (Catlett et al. 2003). If HHKs transmit their signals via Ypd1, a fundamental question arises: How can diverse environmental and developmental signals converge on a single downstream protein yet produce distinct cellular responses? This apparent bottleneck has been termed the “fungal Hpt paradox” (Bourret et al. 2021). One hypothesis to resolve this paradox is that Ypd1 participates in distinct multiprotein complexes, allowing it to function in a context-dependent manner. Another explanation involves alternative splicing, which could generate Ypd1 isoforms that interact with different binding partners. First evidence for the latter mechanism has been reported for *M. oryzae* (Jacob et al. 2015).

A third possibility is that not all HHKs rely on Ypd1 for signal transmission; instead, some may interact with alternative downstream effectors, thereby bypassing Ypd1 entirely (Bourret et al. 2021). Experimental evidence supporting this concept has recently emerged: using recombinant proteins from *Chaetomium thermophilum*, Paredes-Martínez et al. (2024) demonstrated that the REC domains of group III, V, and VI HHKs efficiently phosphorylated Ypd1 *in vitro*, whereas group IV and XI HHKs showed only weak or negligible activity in this assay. Hence, only a subset of HHKs may directly engages Ypd1, while others may signal through yet unidentified target proteins.

Ypd1 is an essential protein in *A. fumigatus* and *A. nidulans* (Schruefer et al. 2021b, Yoshimi et al. 2021) and the conserved His residue at position 89 is crucial for its function (Schruefer et al. 2021b), which is consistent with Ypd1 mediating phosphotransfer in the multistep phosphorelay. Because none of the single HHK deletions in *A. fumigatus* is lethal (Chapeland-Leclerc et al. 2015), maintenance of viability through repression of the HOG pathway requires phosphorylation of Ypd1 by multiple HHKs.

In *A. fumigatus*, exposure to fludioxonil and deletion of *ypd*1 are lethal unless both branches of the HOG pathway, are simultaneously inactivated (Schruefer et al. 2021b). Hence, the combined response of SakA and Skn7 is necessary and sufficient for the deleterious activity that results from a loss or a permanent dephosphorylation of Ypd1. How fludioxonil impacts on the phosphorylation of Ypd1 will be discussed in one of the following chapters.

Apart from TcsC and TcsB, the phytochrome FphA is the best candidate for a HHK interacting with Ypd1. Using bimolecular fluorescence complementation and coimmunoprecipitation assays, Yu et al. (2016) demonstrated a direct physical interaction between FphA and Ypd1 in *A. nidulans* and thereby established a molecular link between light sensing and the multistep phosphorelay system. More recent data obtained with *A. flavus* demonstrate Ypd1 phosphorylation by FphA using phospho-proteome analysis and *in vitro* phosphorylation experiments. Deletion of *fph*A in *A. flavus* results in reduced mycelial growth and a markedly diminished production of aflatoxin 1 thereby providing a link between the HOG pathway and mycotoxin production (Tian et al. 2025).

In conclusion, all these findings highlight Ypd1 as the central element of the multistep phosphorelay. At the same time, they emphasize the remarkable plasticity of fungal signaling networks, in which diverse HHKs can converge on Ypd1 or, potentially, engage distinct pathways to modulate downstream stress responses. Identifying the relevant components and elucidating how the various signaling threads are interwoven will be crucial to understand HHK-mediated signaling and its evolution in filamentous fungi. In *A. fumigatus* and many other fungi, Ypd1 is an essential, fungal-specific protein and therefore an ideal drug target. From an evolutionary perspective it is surprising that these fungi have not implemented functional redundant mechanisms to avoid this dangerous vulnerability.

## Group III HHKs as sensor proteins

The defining function of the HOG pathway is adaptation to hyperosmotic stress. In baker’s yeast, this is mediated by the essential group VI HHK Sln1p. Filamentous fungi possess orthologous group VI HHKs; they are not essential in *A. fumigatus* and many other fungi (Yamada-Okabe et al. 1999, Yoshimi et al. 2005, Du et al. 2006) and are often dispensable for osmoadaptation. Instead, this function is commonly assumed by a group III HHK that exerts primary control over the HOG pathway.

This organization is exemplified in *A. fumigatus*. The group III HHK TcsC is essential for adaptation to hyperosmotic stress (McCormick et al. 2012), whereas the group VI HHK TcsB is not required for this response (Chapeland-Leclerc et al. 2015). TcsC-dependent signaling relies on SakA and both response regulators, SskA and Skn7. Single deletion mutants of all three genes display increased sensitivity to high salt concentrations, yet exhibit wild-type-like growth on medium supplemented with 2 M sorbitol. In contrast, the Δ*sak*AΔ*skn*7 double mutant and the Δ*tcs*C mutant are severely impaired under this condition, demonstrating that both response regulators contribute, at least partially redundantly, to TcsC-mediated osmoadaptation (Schruefer et al. 2021a).

Both Sln1p in *S. cerevisiae* and TcsC in *A. fumigatus* regulate osmoadaptation, but their N-terminal sensor modules differ significantly and both proteins sense stress via different mechanisms. Sln1p is an essential protein and its kinase activity controls the downstream MAP kinase cascade (Posas et al. 1996). TcsC is, in contrast, not essential in *A. fumigatus* (McCormick et al. 2012), but it would be if it were the only HHK that phosphorylates Ypd1. Hence, more *A. fumigatus* HHKs have to be involved and TcsB (the ortholog of Sln1p) is a prime but not proven candidate for this function. However, according to our unpublished data, a Δ*tc*sC*tcs*B^tet-on^ double mutant of *A. fumigatus* is viable under noninducing conditions, indicating that additional HHKs must be involved in the phosphorylation of Ypd1.

The observation that hyperosmotic stress responses are governed by either a type III or a type VI HHK raises an intriguing evolutionary question: did the regulatory architecture observed in *S. cerevisiae* emerged through reductive evolution from an ancestrally larger HHK repertoire, or did filamentous fungi instead expanded their set of HHKs from an ancestral state containing only a single group VI HHK? This aspect will be discussed in one of the following chapters.

## Group III HHKs as antifungal targets

As therapeutic targets, group III HHKs are of particular interest because they are absent from mammals and their aberrant activation disrupts fungal osmotic balance, which often results in cell death (Shor and Chauhan 2015). Certain bacteria learnt to exploit this vulnerability: *Pseudomonas pyrrocinia* strains produce pyrrolnitrin, a secondary metabolite that lethally activates group III HHKs to suppress fungal competitors (Arima et al. 1964). A photostable derivative, fludioxonil, has been used as an agricultural fungicide for decades to control plant pathogens like *B. cinerea* (Kilani and Fillinger 2016). Other structurally unrelated compounds, such as the dicarboximides iprodione and vinclozolin, also act through TcsC and related group III HHKs to induce a similar outcome (Hagiwara et al. 2013, Wiedemann et al. 2016). Hence, group III HHKs can be aberrantly activated by diverse substances to harm filamentous fungi.

Antifungal activity of fludioxonil was described for many fungal species, but the most detailed data on the cellular changes that are caused by this agent stem from studies on *A. fumigatus*. Exposure to fludioxonil (or pyrrolnitrin) induces a broad spectrum of cellular abnormalities, including hyphal swelling, hyperseptation, closure of all septal pores, mitotic dysregulation, and extensive cell wall remodeling (McCormick et al. 2012, Wiedemann et al. 2016, Böhmer et al. 2020). All these phenotypes are aspects of a process that finally results in lysis. Hence, agents that target TcsC have a deleterious impact on *A. fumigatus* and therefore represent promising lead compounds for the development of novel therapeutics to combat infections caused by this major fungal pathogen.

Fludioxonil is also effective against several rare but clinically significant filamentous pathogens, such as *Lichtheimia, Rhizopus*, and *Lomentospora* (*Scedosporium*) species (Wiedemann et al. 2016).

Most *Aspergillus* species are sensitive; only *A. terreus* displays intrinsic resistance to fludioxonil but this species is still susceptible to the dicarboximide iprodione, indicating subtle differences in group III HHK activation through these agents (Wiedemann et al. 2016). The antifungal activity of fludioxonil is more pronounced in *A. fumigatus* than in *A. nidulans* (Wiedemann et al. 2016). This difference correlates with two notable observations. First, loss of group III HHKs leads to increased resistance to the cell wall-perturbing agents Calcofluor white and Congo red in *A. fumigatus*, whereas comparable effects are not found with a corresponding *A. nidulans* mutant. Second, fludioxonil treatment induces shedding of surface-exposed galactomannan and β-1,3-glucan in *A. fumigatus*, but not in *A. nidulans* (Böhmer et al. 2020). Together with evidence suggesting that cell wall remodeling contributes to the antifungal activity of fludioxonil in *A. fumigatus* (Schruefer et al. 2023), these findings may provide an explanation for the comparatively lower sensitivity of *A. nidulans*.

A transcriptional analysis revealed that fludioxonil elicits extensive changes in gene expression in *A. fumigatus* and that this requires TcsC. The response to fludioxonil affects not only genes associated with cell wall biogenesis and remodeling, but triggers a multifaceted stress response also encompassing genes linked to ribosomal function, peroxisome activity, iron homeostasis, and oxidative stress (Schruefer et al. 2023). The fungicidal effect likely results from a combination of increased internal osmotic pressure and an aberrant cell wall architecture. The resulting swelling at a certain point exceeds the cell wall’s mechanical limits, leading to rupture and cytoplasmic leakage (Schruefer et al. 2021b).

Characterization of a series of *A. fumigatus* deletion mutants revealed that Skn7 plays a particularly important role in the response to fludioxonil. Although a Δ*skn*7 mutant exhibits increased resistance compared with the wild type or a Δ*sak*A mutant strain, it remains more susceptible than the fully resistant Δ*tcs*C single or the Δ*skn*7Δ*sak*A double mutant. The importance of Skn7 is also supported by transcriptomic analyses demonstrating that the extensive transcriptional response to fludioxonil in the wild type is markedly reduced in the Δ*skn*7 mutant and almost completely abolished in the Δ*tcs*C mutant. Notably, the genes that are differentially regulated in a Skn7-dependent manner are often involved in cell wall organization, and the Δ*skn*7 mutant largely lacks the fludioxonil-induced changes in cell wall glycostructures (Schruefer et al. 2021a). These findings indicate that Skn7 plays a major role in this cell wall remodeling, which may compromise the stability of the cellular envelope and may thereby contribute to the lethal action of fludioxonil.

The production of pyrrolnitrin by *Pseudomonas* species highlights that bacteria identified group III HHKs as important target structures. Another very promising target is Ypd1, but this is no universally applicable strategy since Ypd1 is not essential in *M. oryzae* and *C. albicans* (Mavrianos et al. 2014, Jacob et al. 2015) and reduced expression of Ypd1 in *C. albicans* can even enhance pathogenicity by promoting hyphal growth (Day et al. 2017). However, a recent study showed that coculture of *C. albicans* with either *Escherichia coli* or *Pseudomonas aeruginosa* results in the production of a soluble bacterial factor that targets Sln1 and induces cell wall reorganization. As a consequence, *Candida* cells increased in size and displayed higher mannan content as well as increased surface exposure of β-1,3-glucan (Davis et al. 2026). These findings provide evidence for another bacteria-induced antifungal activity on the HOG pathway. It is therefore conceivable that a variety of bacterial effector molecules exist that target HHKs or Ypd1 to trigger deleterious activation of the HOG pathway. Further research is clearly needed to identify such compounds and to elucidate their mechanisms of action. A better understanding of interkingdom antagonism represents an important avenue for discovering novel effector molecules and a prerequisite for their therapeutic exploitation.

## The role of HAMP domains in Group III HHK signaling

The N-terminal sensing module of group III HHKs consists of multiple HAMP domains, whose number varies among fungi, often odd in yeasts and even in filamentous molds. The term HAMP derives from protein families comprising this domain, namely histidine kinases, adenylate cyclases, methyl-accepting proteins, and phosphatases. The HAMP domain cluster modulates the activity of the C-terminal signaling module of group III HHKs. Meena et al. (2010) proposed that changes in the dimerization pattern of adjacent HAMP domains determine the protein’s functional state. They also found that a truncated *Debaryomyces hansenii* group III HHK (DhNik1) lacking all five HAMP domains is inactive (Meena et al. 2010). However, expression of a similarly truncated *C. albicans* Nik1 protein caused a severe growth inhibition (El-Mowafy et al. 2013). Both constructs were analysed after heterologous expression in *S. cerevisiae*; although contradictory, they demonstrate that the HAMP cluster regulates the C-terminal signaling module.

Certain HAMP domains are of particular functional importance. From the five HAMP domains of DhNik1, the penultimate HAMP_4_ domain is essential for sensing of hyperosmotic stress (Meena et al. 2010). In *A. fumigatus*, TcsC harbors six HAMP domains and a complete removal of all of them results in a lethal phenotype identical to that triggered by fludioxonil (Spadinger and Ebel 2017). This indicates that, in a resting state, the HAMP cluster prevents a lethal activation of the HOG pathway. The aberrant activity of the truncated protein is suppressed when the HAMP_6_ domain is retained, indicating that this domain exerts a negative regulatory effect on the neighboring signaling module. Taken together, these observations indicate that some HAMP domains are pivotal for the activity and regulation of group III HHKs. However, the functional hierarchy within the HAMP cluster appears to vary among species, with the number of HAMP domains exerting a major influence. Collectively, these findings support a model in which group III HHKs are active kinases under resting conditions and shift into a distinct activity in response to environmental stress or antifungal agents.

**Figure 5:**
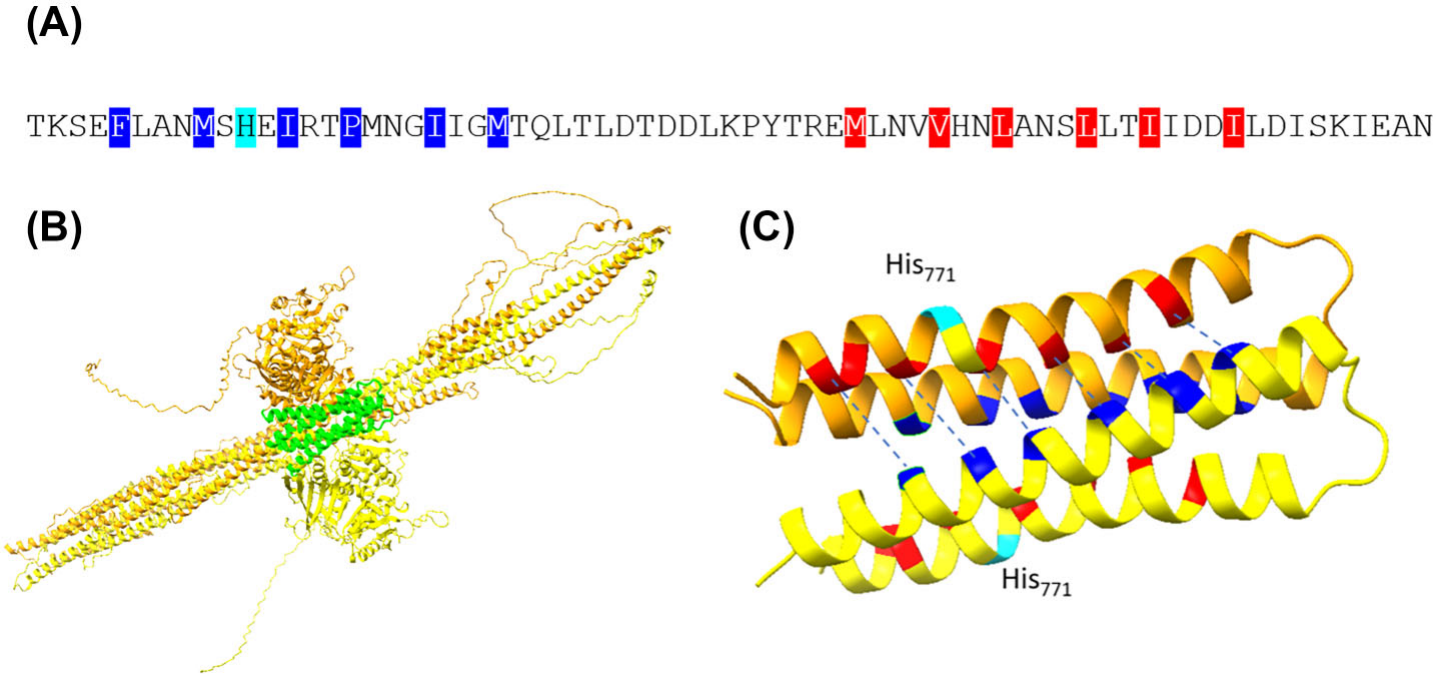
The dimerization motif in TcsC. Panel A shows the amino acid sequence of the HisKA domain of TcsC. The residues of the dimerization interface are indicated in red and blue. His_771_ is highlighted in turquoise. Panel B shows the structure of a TcsC dimer predicted by AlphaFold. The two monomers are stained in yellow and orange, their HisKA domains are colored in green. Panel C shows only the two HisKA domains (in yellow and orange). The amino acids of the putative dimerization interface are indicated in blue and red, the His_771_ residue in turquoise. The dashed lines indicate potential interactions.

Interestingly, a truncated version of TcsC lacking HAMP domains 1 to 5 has a dominant-negative effect when co-expressed with full-length TcsC in *A. fumigatus* (Spadinger and Ebel 2017). This observation suggests that group III HHKs form homodimers, analogous to bacterial two-component systems (Tomomori et al. 1999). In bacteria, such dimerization is mediated by the DHp (dimerization and histidine phosphotransfer) domain. Similar motifs are also predicted in their fungal counterpart, the HisKA domain. A conserved dimerization interface within the HisKA domain exists in TcsC (Fig. 5) and other group III HHKs, such as Drk1 and DhNik1. This motif is likewise present in ten additional HHKs of *A. fumigatus*, yet notably absent in Fhk4, TcsB, and its *S. cerevisiae* counterpart Sln1p (Table 1). The conservation of this motif in bacterial two-component systems and many fungal HHKs suggests that it represents an ancient and fundamental feature of these signaling molecules that enables a dimerization-dependent regulation

The predicted dimerization motif consists of two blocks of six amino acids, with each residue within a block separated by two to three intervening residues. Remarkably, the conserved His residue of the HisKA domain is part of this motif (His_771_ of TcsC; Fig. 5). It is therefore very likely that phosphorylation of this residue has a strong influence on dimer formation and thereby controls the activity of the protein.

The fact that only group III HHKs respond to agents, such as fludioxonil, pyrrolnitrin, or iprodione suggest that these compounds interfere directly or indirectly with the HAMP cluster. Sequencing of the group III HHK Bos1 from *B. cinerea* isolates that acquired resistance to fludioxonil and/or iprodione revealed that many mutations affected hydrophobic residues that perturbed the helical structures of HAMP domains 2–4 (Fillinger et al. 2012). While this suggests a direct interaction of the antifungals with particular HAMP domains, other evidence points to a more indirect interaction. Brandhorst et al. (2019) showed that fludioxonil influence the activity of the triosephosphate isomerase of *Blastomyces dermatitidis* and thereby causes methylglyoxal stress that alters the activity of the group III HHK Drk1.

### Fludioxonil most likely disrupts Ypd1 phosphotransfer by driving a kinase-to-phosphatase transition in group III HHKs

**Figure 6:**
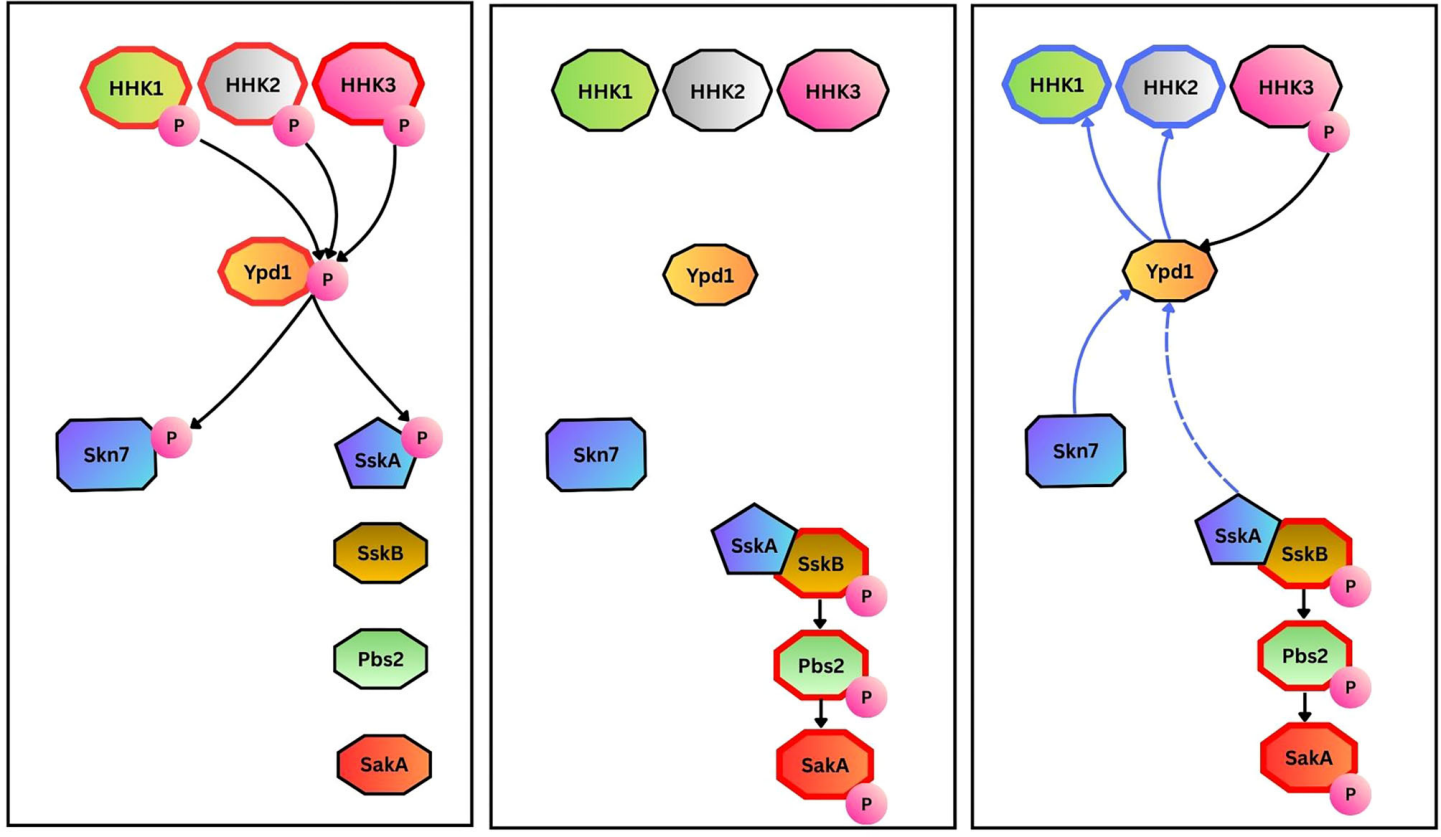
Model of two possible modes of HOG pathway activation in *A. fumigatus*. The unstressed state is shown on the left. Activation of the HOG pathway resulting from loss of HHK kinase activity is shown in the center, whereas activation mediated by a switch of HHKs toward phosphatase activity is illustrated on the right. How many HHKs interact with Ypd1 remains an unsolved question. TcsC and FphA are most likely involved. Phosphorylation reactions are indicated by black arrows, and phosphorylated proteins are marked with a red “P.” The flow of phosphoryl groups resulting from phosphatase activity of one or more HHKs is indicated by blue arrows. Whether the phosphoryl group of SskA is accessible remains unclear, as phosphorylation of Ssk1p by Ypd1p in *S. cerevisiae* has been reported to be irreversible (Janiak-Spens et al. 2005). Protein with kinase activity are outlined in red, whereas proteins with phosphatase activity are outlined in blue.

In *A. fumigatus* a fludioxonil-like phenotype can be genetically elicited in three different ways: by deletion of Ypd1, by mutation of its conserved His_89_ residue, or by expression of a truncated TcsC variant lacking all HAMP domains. These observations indicate that inhibition of Ypd1-mediated phosphotransfer constitutes the antifungal mechanism of phenylpyrrole compounds (Schruefer et al. 2021b, Yoshimii et al. 2021). Notably, the activity of HAMP-deficient TcsC abolishes Ypd1-dependent phosphotransfer, albeit through a mechanism that appears mechanistically distinct from the stress-induced loss of kinase activity described for Sln1p. If fludioxonil would just stall the kinase activity of TcsC, loss of *tcs*C should be lethal, but a Δ*tcs*C mutant grows normally (McCormick et al. 2012). Hence, if TcsC phosphorylates Ypd1 under resting conditions (basal activity), it must switch to an alternative mode of activity upon stress or chemical activation (induced activity) (Fig. 6).

It is generally assumed that group III HHKs possess a kinase activity under resting conditions to phosphorylate Ypd1 and thereby to maintain the HOG pathway in an inactive state. Upon exposure to hyperosmotic stress or certain antifungal agents, HHKs undergo a functional transition. Two mechanistic scenarios can be envisioned: (i) a change in substrate specificity, for example phosphorylation of an alternative His residue on Ypd1, or (ii) a switch in enzymatic activity, e.g. from kinase to phosphatase. Multiple lines of evidence support the second model, indicating that stress conditions promote a kinase-to-phosphatase transition, thereby driving dephosphorylation of the phosphorelay and activation of downstream HOG signaling.

Bacterial two-component systems provide a precedent for this activity switching. A dual kinase/phosphatase activity of ArcB enables adaptation of *E. coli* to an anaerobic environment (Iuchi 1993). The *lux* system of the bioluminescent marine bacterium *Vibrio harveyi* is another well-studied system. The hybrid sensor proteins LuxQ and LuxN exhibit dual functionality, acting as either kinase or phosphatase depending on cell density (Parkinson and Kofoid 1992). In the uninduced state, LuxN phosphorylates the Hpt protein LuxU and only slowly dephosphorylates phosphorylated LuxU. When the quorum-sensing molecule AI-1 is present, LuxN’s kinase activity is inhibited while its phosphatase function is retained, effectively reversing the net phosphotransfer flow (Timmen et al. 2006).

In a biochemical assay, Azuma et al. (2007) demonstrated that the bacterial histidine kinase ArcB can phosphorylate the *A. nidulans* phosphotransfer protein YpdA. Remarkably, they also described “retrograde phosphorylation,” whereby YpdA transfers the phosphoryl group back to the HHK FphA. Evidence for a kinase-to-phosphatase transition in fungal group III HHKs was first obtained for Drk1 of *B. dermatitidis. In vitro*, a recombinant REC domain of Drk1 displayed intrinsic phosphatase activity toward Ypd1, and this activity depended on the conserved Asp residue at position 1140 within the REC domain (Lawry et al. 2017). Full-length Drk1, by contrast, displayed intrinsic kinase activity in its resting state, suggesting that environmental cues toggle Drk1 between these two opposing states.

A model in which group III HHKs switch from kinase to phosphatase activity (Fig. 6) provides an elegant framework for explaining the function of TcsC in *A. fumigatus* and likely also of homologous HHKs in other filamentous fungi. Under nonstress conditions, TcsC functions as a kinase, thereby contributing to the phosphorylation of Ypd1. In a Δ*tcs*C mutant, this kinase activity is lost, requiring other HHKs to maintain sufficient Ypd1 phosphorylation to prevent inappropriate activation of downstream signaling components.

Upon stress, however, TcsC cannot simply continue to function as a kinase. Nor can its kinase activity merely be switched off, as proposed for *S. cerevisiae* Sln1p, because this situation would resemble that of a nonstressed Δ*tcs*C mutant, which is unable to adapt to hyperosmotic stress or respond to fludioxonil. Instead, a stress-induced switch from kinase to phosphatase activity would provide a plausible mechanism to actively promote dephosphorylation of Ypd1, and consequentially, an activation of the HOG pathway.

Although this dual-activity model offers a compelling explanation for the available genetic and physiological data, direct experimental evidence for phosphatase activity of group III HHKs remains limited. Further biochemical studies are therefore required to validate this hypothesis. Moreover, the dual-activity model raises the possibility that group VI HHKs may possess a comparable functional plasticity, a hypothesis that warrants experimental investigation.

For a mechanistic understanding, it will be essential to determine whether the conserved His and Asp residues within the HisKA and REC domains of group III HHKs are required for both enzymatic activities. In bacterial two-component systems, kinase activity depends on the conserved His within the Dhp domain. In contrast, this residue is not absolutely required for phosphatase activity; however, it can promote dephosphorylation by accelerating the intrinsic instability of the Asp-phosphorylation and is therefore often necessary for full catalytic efficiency (Hsing and Silhavy 1997, Freeman et al. 2000, Willett and Kirby 2012).

Conflicting data exist regarding the functional role of the conserved His residue within the HisKA domain of fungal HHKs. In MoHik1 of *M. oryzae*, this residue is essential for osmotic stress signaling, but not for the antifungal activity of fludioxonil (Bersching and Jacob 2021), but other studies have reported divergent findings. Truncated variants of TcsC from *A. fumigatus* and DhNik1 from *D. hansenii* exhibit lethal activity that phenocopies fludioxonil exposure and likely reflects constitutive phosphatase activity. In these truncated proteins, the lethal activity depends on the conserved His residue within the HisKA domain (Spadinger and Ebel 2017) and in DhNik1, it additionally requires the conserved Asp residue of the REC domain (Sasan et al. 2025). Structural modeling of native and truncated DhNik1 suggests that the truncation prevents a proper closure of the ATP lid, thereby impairing nucleotide retention and favoring phosphatase over kinase activity (Sasan et al. 2025).

Work on Drk1, the group III HHK of *B. dermatitidis* demonstrated that an isolated REC domain dephosphorylates Ypd1 *in vitro* (Lawry et al. 2017), but the isolated REC domain of the group III HHK from *C. thermophilum* phosphorylated Ypd1 in a similar assay (Paredes-Martínez et al. 2024). Hence, the question of whether isolated REC domains possess intrinsic phosphatase activity, or whether this activity requires additional parts of the protein, remains unresolved.

The study of Sasan et al. (2025) provides another intriguing insight. As mentioned above, these authors found that a truncated sensing module of DhNik1 has a lethal activity. Notably, they observed a similar activity for a DhNik1 harboring a mutated N-terminal sensing module. Fusion of this mutated sensing module to the signaling module of Sln1p of *S. cerevisiae* likewise resulted in a deleterious activity suggesting that the signaling module of Sln1p can also adopt a phosphatase activity.

A more systematic functional analysis of isolated signaling modules and REC domains from multiple group III HHKs will be an important next step in this field. Such analyses should include HHKs from other groups, particularly group VI (Sln1p/TcsB) and group VIII (FphA).

If the model described above is correct, multiple HHKs cooperate in filamentous fungi to phosphorylate Ypd1 under nonstress conditions, thereby keeping the HOG pathway inactive. Certain stress conditions are detected only by specific HHKs. Upon sensing their respective stimuli, these kinases switch functionally to phosphatases and can dephosphorylate the multistep phosphorelay, even though other HHKs remain active as kinases. Activation of the pathway therefore depends on whether the phosphatase activity predominates.

For example, FphA and TcsC sense light and hyperosmotic stress, respectively. Following a shift to a hyperosmotic environment, TcsC may acquire phosphatase activity, whereas FphA remains active as a kinase. These activities have opposing effects on the phosphorylation state of Ypd1; however, if phosphatase activity prevails, the HOG pathway becomes activated.

Initially the HOG pathway of *S. cerevisae* was thought to become activated if the kinase activity of Sln1p is stalled. In filamentous fungi, switch of certain HHKs to phosphatase activity seems to govern the activation of the HOG pathway. The findings of Sasan et al. (2025) now raise the possibility that Sln1p may also be capable of adopting a phosphatase activity.

## Evolution of the repertoire of HHKs

Comparative genomic and phylogenetic analyses of the HOG pathway indicate that the core MAP kinase cascade (MAPKKK–MAPKK–MAPK) is remarkably conserved, whereas HHKs exhibit substantially greater variability (Nikolaou et al. 2009, Wu et al. 2010) reflecting a specialization of these receptor proteins. The individual requirements in each fungal species led to the evolution of a distinct set of HHKs tailored to the specific needs of this fungus.

Evolutionary reconstructions suggest that early fungi possessed multiple HHKs and that filamentous fungi have largely retained or expanded this repertoire (Hérivaux et al. 2018, Catlett et al. 2003). In contrast, budding yeasts of the Saccharomycotina lineage have experienced progressive loss of HHKs (Hérivaux et al. 2018). Within the Saccharomycotina, two groups can be distinguished: species belonging to basal lineages, the WGD clade, the methylotrophs, and the *Wickerhamomycetaceae* retain group III HHKs, whereas the *Saccharomycodaceae*, the Kluyveromyces/Lachancea/Eremothecium clade, the Zygosaccharomyces/Torulaspora clade, and the post-WGD clade lack group III and other HHKs (Hérivaux et al. 2018). In these latter taxa, which are more distantly related to the ancestral Saccharomycotina precursor, the HHK repertoire is streamlined and centered on the essential sensor Sln1p. In conclusion, these data suggest that several HHKs, including those of group III and VI, were initially present and that evolutionary processes tended either toward an expansion or a narrowing of this repertoire.

Although group III and VI have both been implicated in the adaptive response to hyperosmotic stress, their sensory apparatus is structurally distinct: group III HHKs contain multiple HAMP domains, whereas group VI HHKs detect turgor changes via their extracellular part that lacks any recognizable domains.

Whether HHKs of group III or VI represent the more ancestral hyperosmotic stress receptor, or whether both initially cooperated in stress sensing remains unclear. In *M. oryzae*, deletion of the group VI HHK increased sensitivity to salt stress, whereas deletion of the group III HHK impaired growth in the presence of high concentrations of sorbitol (Jacob et al. 2014). This suggests that functional specialization can occur when both receptor types are present. Although high salt and high sorbitol are commonly used to induce hyperosmotic stress, they differ substantially: salt imposes ionic and osmotic stress, whereas sorbitol causes only osmotic stress (Nikolaou et al. 2009, Blomberg 2022). Dedicated receptors for these distinct stress modalities would therefore be plausible.

In a study focusing on pathogenic fungi belonging to the Ascomycota, Basidiomycota, and Mucormycotina, group III HHKs were found in all 24 species, but only half of them harbored also a group VI HHK (Defosse et al. 2015). A broader genomic survey of 91 fungal species identified also substantially more group III (123) than group VI (42) HHKs; notably, group VI HHKs were restricted to Ascomycota, whereas group III HHKs were also found in Basidiomycota and early-diverging fungi (Mina et al. 2024). Thus, many fungal species lack group VI HHKs but are nevertheless able to tolerate hyperosmotic stress (Krantz et al. 2006). In some fungi, including *S. cerevisiae*, the response to hyperosmotic stress can be mediated by the Sho1 branch of the HOG pathway. In other fungi, however, Sho1 homologs do not contribute to osmoadaptation. In these species, group III HHKs may functionally compensate for the absence of a group VI HHK.

In *A. nidulans*, the group VI HHK TcsB can activate the HOG pathway when expressed in *S. cerevisiae* (Furukawa et al. 2002). However, deletion of *tcs*B does not impair the osmotic stress response in *A. nidulans*, whereas loss of the group III HHK NikA confers high sensitivity to salt and sorbitol (Hagiwara et al. 2009). A similar group III dominance has been reported for *A. fumigatus* (McCormick et al. 2012). Thus, although group VI HHKs may retain intrinsic osmosensory capacity, regulatory control of the HOG pathway in *Aspergillus* species was taken over by group III HHKs.

Reconstructing the evolutionary development of these osmosensors is still a major challenge in this field of research. One possible scenario is that *M. oryzae* represents an ancestral state in which group III and group VI HHKs cooperatively mediate adaptation to hyperosmotic stress, with group III responding primarily to sorbitol and group VI to salt stress. In *Aspergillus* and other filamentous fungi, group III HHKs are the principal osmosensors. Group VI HHKs are often still present but of unclear physiological relevance. In some filamentous fungi, this process has intensified to the point of a complete loss of group VI HHKs (Mina et al. 2024). *Saccharomyces cerevisiae* and related species may have followed an opposite path and a reduced HHK repertoire forced Sln1p to acquire full responsiveness for hyperosmotic stress.

As this model is based on a limited dataset, comparative studies across the fungal kingdom will be necessary to elucidate the evolutionary processes that have shaped the repertoire of sensor proteins involved in hyperosmotic stress sensing.

## The spatial dimension of the multistep phosphorelay

The intracellular environment of eukaryotic cells is highly compartmentalized, comprising numerous specialized organelles that define distinct signaling niches (Gabaldón and Pittis 2015). Within this architectural framework, the two response regulators of the multistep phosphorelay occupy discrete subcellular domains: Ssk1/SskA is confined to the cytosol, where it regulates the MAP kinase cascade, whereas Skn7 is efficiently targeted to the nucleus (Schruefer et al. 2021b, Raitt et al. 2000, Lu et al. 2003), consistent with its role as a transcription factor. Remarkably, the localization pattern of both response regulators is not modulated by their activation state.

Efficient signal transduction to both response regulators requires Ypd1 to shuttle between the cytoplasm and the nucleus. Owing to its small molecular mass, Ypd1 can freely diffuse through nuclear pores (Schruefer et al. 2021b, Lu et al. 2003). Group VI HHKs, such as Sln1p in *S. cerevisiae* or TcsB in *A. fumigatus*, contain two transmembrane helices that anchor them to the plasma membrane (Fig. 2). By contrast, most other HHKs are predicted to be cytoplasmic proteins (Chapeland-Leclerc et al. 2015). The sole exceptions identified so far are FphA, FphB, and TcsC, which exhibits a predominant nuclear localization under nonstress conditions (Vincek et al. 2024, Leister et al. 2025).

The subcellular distribution of TcsC is dynamic and changes in response to environmental cues. Following exposure to fludioxonil, TcsC gradually relocates from the nucleus to the cytoplasm within ~40 min (Vincek et al. 2024). Two findings suggest that the relatively slow transition of TcsC reflects an impaired nuclear import rather than a directed export of activated proteins. First, activation of the HOG pathway by fludioxonil, indicated by the translocation of SakA to the nucleus, is much faster and occurs within 5 min (Vincek et al. 2024). Second, in the absence of stress, TcsC undergoes a continuous nucleocytoplasmic shuttling, which takes ~40 min for a complete replacement.

An intriguing possibility is that the homodimerization of activated group III HHKs may interfere with nuclear import, thereby contributing to the stress-induced cytoplasmic retention of TcsC. Further experiments are clearly needed to understand the physiological relevance of the dynamic localization of TcsC and to investigate whether similar localization patterns are also found for other HHKs.

Collectively, these findings underscore that the spatial dimension of the multistep phosphorelay adds another layer of complexity to the HOG pathway. The interplay between HHK localization, Ypd1 mobility, and compartment-specific response regulators offers a versatile mechanism for integrating diverse environmental inputs into appropriate cellular responses.

## The HOG pathway as part of a complex signaling network

The HOG pathway is one of the principal stress response systems in fungal cells (Bahn et al. 2007). Rather than functioning in isolation, these pathways operate within a highly interconnected network that integrates diverse environmental and physiological inputs to generate tailored responses. The response regulator Skn7 exemplifies this complexity: it participates in the multistep phosphorelay of the HOG pathway and fulfills an independent role in the oxidative stress response (Schruefer et al. 2021a, He et al. 2009, Mulford and Fassler 2011). Moreover, Skn7 contributes to cell wall remodeling in both *S. cerevisiae* and *A. fumigatus* (Schruefer et al. 2021a, Fassler and West 2011), reflecting overlapping activities with the cell wall integrity pathway. Skn7p is a transcription factor (Morgan et al. 1995) and phosphorylation of its REC domain is important for its cell wall-related activity, but not for its response to oxidative stress implicating different downstream effector mechanisms (Li et al. 2002, Fassler and West 2011).

The concept that group III HHKs switch from a kinase to a phosphatase activity upon activation implies that fludioxonil-induced cell wall remodeling would result from dephosphorylation of Skn7. In *S. cerevisiae*, phosphorylation of Skn7 activates its function as a transcription factor (Li et al. 2002); accordingly, stress-induced dephosphorylation would be expected to reduce expression of Skn7 target genes. However, transcriptional profiling of *A. fumigatus* revealed the opposite: fludioxonil induces a strong and Skn7-dependent upregulation of numerous known or putative cell wall-associated genes (Schruefer et al. 2023). This apparent discrepancy suggests that the regulatory mechanism governing Skn7 activity in filamentous fungi may differ from that in yeast, or that the underlying pathways are more complex than previously anticipated.

A close functional interconnection also exists between the HOG and the cell wall integrity pathway. This is reflected in the ability of group III HHKs to promote both osmotic adaptation and cell wall remodeling (Mattos et al. 2020, Fuchs and Mylonakis 2009). At the experimental level, this was demonstrated by the fact that the related MAP kinases MpkA, MpkC, and SakA directly interact and collaborate in the response to cell wall stress (Manfiolli et al. 2019, Mattos et al. 2020).

## Conclusions and outlook

Despite substantial progress, the biological roles of many fungal HHKs remain incompletely understood. Their remarkable diversity in structure, potential dimerization, localization, and regulatory activity indicates that HHKs are versatile proteins whose functions extend far beyond osmoadaptation.

The frequent absence of discernible phenotypes in single HHK mutants of different fungi implies functional redundancy and overlapping activities among different members of this protein family. Understanding how HHKs integrate environmental cues, coordinate with other signaling pathways, and regulate subcellular compartmentalization represents one of the key frontiers in fungal cell biology.

Future studies should move beyond descriptive analyses and address the mechanistic principles that determine HHK activity states. In particular, defining the structural and biochemical determinants that dictate kinase versus phosphatase output, mapping compartment-specific interaction networks, and tracing the evolutionary diversification of these signaling modules across fungal lineages will be essential to fully understand HHK function.

From an applied standpoint, HHKs and Ypd1 represent two particularly attractive antifungal targets. They are restricted to fungi and occupy pivotal regulatory nodes that integrate environmental stress cues with adaptive responses. The antifungal activity of pyrrolnitrin underscores that bacterial competitors have long exploited vulnerabilities within fungal signaling systems. It is therefore reasonable to assume that microbial secondary metabolism harbors a broader repertoire of Ypd1- or HHK-targeting molecules, representing an underexplored resource for the discovery of novel antifungals.
